# Safety, tolerability, pharmacokinetics and effect on serum uric acid of the myeloperoxidase inhibitor AZD4831 in a randomized, placebo‐controlled, phase I study in healthy volunteers

**DOI:** 10.1111/bcp.13855

**Published:** 2019-02-18

**Authors:** Li‐Ming Gan, Maria Lagerström‐Fermér, Hans Ericsson, Karin Nelander, Eva‐Lotte Lindstedt, Erik Michaëlsson, Magnus Kjaer, Maria Heijer, Carl Whatling, Rainard Fuhr

**Affiliations:** ^1^ Early Clinical Development IMED Biotech Unit, AstraZeneca Gothenburg Sweden; ^2^ Department of Molecular and Clinical Medicine, Institute of Medicine Sahlgrenska Academy at the University of Gothenburg Gothenburg Sweden; ^3^ Department of Cardiology Sahlgrenska University Hospital Gothenburg Sweden; ^4^ Cardiovascular, Renal and Metabolism IMED Biotech Unit, AstraZeneca Gothenburg Sweden; ^5^ PAREXEL Early Phase Clinical Unit Berlin Germany

**Keywords:** cardiovascular disease, myeloperoxidase, phase I clinical trial

## Abstract

**Aims:**

Myeloperoxidase activity can contribute to impaired vascular endothelial function and fibrosis in chronic inflammation‐related cardiovascular disease. Here, we investigated the safety, tolerability and pharmacokinetics of the myeloperoxidase inhibitor, AZD4831.

**Methods:**

In this randomized, single‐blind, placebo‐controlled, phase I, first‐in‐human study, healthy men in five sequential cohorts were randomized 3:1 to receive a single oral dose of AZD4831 (5, 15, 45, 135 or 405 mg) or placebo, after overnight fasting. After at least 7 days' washout, one cohort additionally received AZD4831 45 mg after a high‐calorie meal.

**Results:**

Forty men participated in the study (eight per cohort: AZD4831, *n* = 6; placebo, *n* = 2). AZD4831 distributed rapidly into plasma, with a half‐life of 38.2–50.0 hours. The area under the plasma concentration–time curve (AUC) increased proportionally with dose (AUC_0–∝_ slope estimate 1.060; 95% confidence interval [CI] 0.9943, 1.127). Increases in maximum plasma concentration were slightly more than dose proportional (slope estimate 1.201; 95% CI 1.071, 1.332). Food intake reduced AZD4831 absorption rate but did not substantially affect overall exposure or plasma half‐life (*n* = 4). Serum uric acid concentrations decreased by 71.77 (95% CI 29.15, 114.39) and 84.42 (58.90, 109.94) μmol L^−1^ with AZD4831 135 mg and 405 mg, respectively. Maculopapular rash (moderate intensity) occurred in 4/30 participants receiving AZD4831 (13.3%). No other safety concerns were identified.

**Conclusions:**

AZD4831 was generally well tolerated, rapidly absorbed, had a long plasma half‐life and lowered uric acid concentrations after single oral doses in healthy men. These findings support the further clinical development of AZD4831.

**What is already known about this subject**

- Myeloperoxidase activity in blood vessels can contribute to impaired vascular endothelial function and fibrosis.
- Elevated plasma myeloperoxidase levels are associated with increased cardiovascular morbidity and mortality in humans.
- Vessel‐associated myeloperoxidase is a promising therapeutic target for the treatment of chronic inflammation‐related cardiovascular disease.

**What this study adds**

- Single 5–405 mg doses of the orally administered myeloperoxidase inhibitor, AZD4831, showed rapid and dose‐dependent systemic absorption in healthy men.
- AZD4831 was well tolerated, with moderate maculopapular rash as the only identified risk.
- AZD4831 administration decreased serum uric acid concentrations; these findings support the further clinical development of AZD4831.

## 1 INTRODUCTION

Myeloperoxidase is an emerging therapeutic target in the development of new treatments for inflammation‐related cardiovascular diseases. The principal function of myeloperoxidase is to mediate the oxidative killing of microbes in neutrophil phagolysosomes. Some myeloperoxidase is, however, also released into the extracellular space, where its oxidative activity can contribute to tissue damage, fibrosis and impaired vascular endothelial function.^1^, ^2^, ^3^ Extracellular myeloperoxidase binds with high avidity to negatively charged proteoglycans on the surface of vascular endothelial cells.^4^, ^5^ This blood vessel‐associated myeloperoxidase can locally deplete nitric oxide, inhibit vasodilation and mediate the recruitment of circulating leukocytes, with resulting amplification of local inflammation.^2^, ^6^

Long‐term elevation of myeloperoxidase activity may be involved in the development of cardiovascular disease in humans. Leukocyte numbers and blood levels of myeloperoxidase are elevated in patients with coronary artery disease.^7^ In patients with acute coronary syndromes or chronic heart failure, high plasma levels of myeloperoxidase are associated with advanced disease and an increased risk of future cardiovascular events.^8^, ^9^, ^10^ Furthermore, increased incidence of atrial fibrillation is associated with elevated plasma levels of myeloperoxidase in patients with pacemakers.^1^ Conversely, individuals with low myeloperoxidase levels are resistant to inflammation‐induced endothelial dysfunction.^11^ Inhibition of myeloperoxidase is associated with reduced plasma concentrations of uric acid,^12^ a marker of oxidative stress and chronic inflammation. Uric acid levels are elevated in people with cardiovascular disease, and high levels are associated with vascular dysfunction and cardiovascular mortality.^13^, ^14^, ^15^ Inhibiting myeloperoxidase may therefore provide therapeutic benefit in patients with cardiovascular diseases, by limiting impairment of vascular function and the subsequent development of fibrosis.

The canonical enzymatic activity of myeloperoxidase is the oxidation of chloride ions (Cl^−^) in the presence of hydrogen peroxide (H_2_O_2_) to produce hypochlorous acid (HOCl), which mediates the bactericidal effect.^16^ Myeloperoxidase can also oxidize a range of other physiological substrates,^17^ including tyrosine,^18^ melatonin,^19^ serotonin,^20^ xanthine 21 and uric acid.^22^

AZD4831 23 is a novel, potent and selective myeloperoxidase inhibitor, with an in vitro half‐maximal inhibitory concentration (IC_50_) of 0.7 nM for human myeloperoxidase and a value of 65% for human plasma protein binding (E Michaëlsson et al., manuscript in preparation). Unpublished in vitro data show that AZD4831 also inhibits the oxidation of xanthine and uric acid by myeloperoxidase (also confirming previous reports that xanthine and urate are myeloperoxidase substrates^21^, ^22^). Furthermore, AZD4831 does not inhibit xanthine oxidase or the urate transporter urate anion exchanger 1 (URAT1; SLC22A12) in vitro (E Michaëlsson et al., manuscript in preparation).

Here, we report the results from a first‐in‐human, phase I study of the safety, tolerability and pharmacokinetics of single ascending doses of AZD4831 in healthy volunteers. The study also assessed serum uric acid levels as an exploratory pharmacodynamic outcome.

## 2 METHODS

### 2.1 Overview and objectives

This was a randomized, single‐blind, placebo‐controlled, phase I, first‐in‐human study of the safety and tolerability of the myeloperoxidase inhibitor, AZD4831, in healthy male volunteers (ClinicalTrials.gov identifier: NCT02712372). The study was originally designed to include a single ascending dose period and a multiple ascending dose period. Based on results and experience from the single ascending dose period, the multiple ascending dose period was cancelled. Multiple ascending doses in healthy volunteers were later tested in a separate study (ClinicalTrials.gov identifier: NCT03136991; to be reported elsewhere).

The study included two parts. In part A, sequential cohorts of participants received a single oral dose of AZD4831 or placebo under fasting conditions. In part B, one cohort of participants who had already received AZD4831 in part A then received a second single oral dose of AZD4831 under fed conditions.

The primary objective of the study was to evaluate the safety and tolerability of AZD4831 in healthy volunteers. Secondary objectives were to characterize the pharmacokinetics of AZD4831 and to investigate the effect of food intake on the pharmacokinetics. Evaluation of the pharmacodynamics of AZD4831 by assessment of uric acid concentration in serum was an exploratory objective.

### 2.2 Conduct and ethics

The study took place between June 2016 and October 2016 at the PAREXEL Early Phase Clinical Unit in Berlin, Germany. It was conducted in accordance with the principles of the Declaration of Helsinki and the International Conference on Harmonisation and Good Clinical Practice. An independent ethics committee and institutional review board reviewed and approved the study protocol and its amendments. All participants freely gave their written informed consent before starting the study. The study was registered with ClinicalTrials.gov (identifier: NCT02712372).

### 2.3 Participants

Male volunteers, aged 18–50 years, weighing 50–100 kg and with a body mass index (BMI) of 18–29.9 kg m^−2^, were eligible for the study. Key exclusion criteria were: a history or presence of any disease or disorder that might influence study participation or results; a history or presence of any condition known to interfere with the absorption, distribution, metabolism or excretion of drugs, including gastrointestinal, hepatic or renal disease; the presence of infection; and a history or presence of thyroid disease. Volunteers with clinically significant abnormalities in vital signs, laboratory analyses or electrocardiogram findings were excluded from the study, as were those with any clinically significant illness, medical procedure or trauma within the previous 4 weeks.

### 2.4 Study design

#### 2.4.1 Part A: randomized, single‐blind, placebo‐controlled, single ascending dose stage

Participants were sequentially assigned to one of five cohorts (5 mg, 15 mg, 45 mg, 135 mg or 405 mg, each *n* = 8) and randomized 3:1 within each cohort to receive an oral dose of AZD4831 (*n* = 6) or matching placebo (*n* = 2) as a suspension in water (maximum total volume 240 mL). Participants fasted overnight for at least 10 hours before dosing. In each cohort, all safety data from a sentinel pair (AZD4831, *n* = 1; placebo, *n* = 1) up to 24 hours postdose were evaluated before the remaining participants in the cohort received their doses. Participants resided at the study centre until 48 hours postdose and attended a follow‐up visit 7–10 days after dosing.

The maximum safe starting dose of AZD4831, applying US Food and Drug Administration guidance,^24^ was calculated as 1.1 mg kg^−1^ based on 20 mg kg^−1^ per day as the dose at which no adverse effects were observed in a preclinical study in dogs. The 5 mg starting dose was predicted to result in minimal or no pharmacological activity. Dose escalation of up to 3‐fold was permitted between cohorts, up to a maximum of 1250 mg per day. The Safety Review Committee reviewed all available safety and pharmacokinetic data from completed cohorts before deciding the dose for the next cohort. Dose escalation was to be stopped if pharmacokinetic data indicated that the predefined maximum AZD4831 exposure level had been reached or was predicted to be reached or exceeded following an increased dose (maximum plasma concentration [C_max_] of 12.7 μmol L^−1^ and/or area under the plasma concentration–time curve [AUC] of 168 μmol*h L^−1^).

#### 2.4.2 Part B: open‐label, food effect evaluation stage

After a washout period of at least 7 days, participants who had received AZD4831 45 mg in cohort 3 in part A received a second dose of AZD4831 45 mg immediately after a high‐calorie, high‐fat breakfast. This dose was selected by the Safety Review Committee based on all available data for one or more higher doses in part A. Participants once again resided at the study centre until 48 hours postdose and attended a follow‐up visit 7–10 days after dosing.

### 2.5 Pharmacokinetic analyses

#### 2.5.1 Plasma pharmacokinetics

Blood samples were collected for plasma pharmacokinetic analyses predose and at 0.25, 0.5, 1, 1.5, 2, 2.5, 3, 4, 6, 8, 12, 24, 36, 48, 72, 96 and 120 hours postdose for all except the first cohort (5 mg of AZD4831 or placebo). Blood samples were collected only up to 48 hours after dosing for the first cohort; the sampling schedule was amended thereafter to include the later time points. Samples for determination of AZD4831 in plasma were analysed by Covance Laboratories Ltd. (Harrogate, UK). AZD4831 and the stable labelled internal standard [^13^C_3_^15^N_2_]AZD4831 were extracted from plasma by liquid–liquid extraction and analysed by liquid chromatography, followed by tandem mass spectrometry (LC–MS/MS). This method was validated prior to sample analysis in the range 2–2000 nmol L^−1^ in plasma, using a 25 μL sample aliquot.

Accuracy and precision were determined using quality‐control (QC) samples of AZD4831 at concentrations of 2, 6, 70, 800, 1600 and 20 000 nmol L^−1^ in plasma. Inter‐run accuracy and precision were in the ranges of 97.0–102.7% and 2.6–12.0%, respectively. At a minimum, each analytical run included a calibration curve, a matrix blank, a control zero sample (matrix blank containing internal standard), a reagent blank and duplicate QC samples at three concentrations within the calibration range. To demonstrate acceptable in‐study performance, incurred sample reproducibility analyses were performed during the study. Of the 60 samples tested, 58 (96.7%) were within 20% of the mean of the two values. All plasma samples were analysed within the known stability period.

Plasma pharmacokinetic endpoints were: observed C_max_; time to C_max_ (*t*_max_); AUC from time 0 to 24 hours postdose (AUC_0–24_), to time of last quantifiable analyte concentration (AUC_0–*t*_) and extrapolated to infinity (AUC_0–∞_); half‐life associated with terminal slope (λz) of a semi‐logarithmic concentration–time curve (*t*_1/2λz_); apparent clearance (CL/F); and apparent volume of distribution (V_z_/F).

#### 2.5.2 Urinary pharmacokinetics

Urine was collected for pharmacokinetic analyses before dosing (spot sample) and 0–3, 3–6, 6–9, 9–12, 12–24, 24–36 and 36–48 hours after dosing (pooled samples). Samples for determination of AZD4831 in urine were analysed by Covance Laboratories Ltd. (Harrogate, UK). AZD4831 and the stable labelled internal standard [^13^C_3_^15^N_2_]AZD4831 were prepared from urine by sample dilution and analysed by LC–MS/MS. This method was validated prior to sample analysis in the range 20–20 000 nmol L^−1^ in urine, using a 25 μL sample aliquot.

Accuracy and precision were determined using QC samples of AZD4831 at concentrations of 20, 60, 1000, 16 000 and 160 000 nmol L^−1^ in urine. Inter‐run accuracy and precision were in the ranges of 93.7–105.0% and 0.6–6.3%, respectively. At a minimum, each analytical run included a calibration curve, a matrix blank, a control zero sample (matrix blank containing internal standard), a reagent blank and duplicate QC samples at three concentrations within the calibration range. To demonstrate acceptable in‐study performance, incurred sample reproducibility analyses were performed during the study. Of the 24 samples tested, 23 (95.8%) were within 20% of the mean of the two values. All urine samples were analysed within the known stability period.

Urinary pharmacokinetic endpoints were: cumulative amount of AZD4831 excreted unchanged in urine; cumulative fraction of dose excreted unchanged in urine; and renal clearance.

### 2.6 Safety and tolerability outcomes

Safety assessments included adverse event (AE) monitoring, vital signs monitoring (systolic and diastolic blood pressure, pulse rate), electrocardiography, physical examinations and laboratory assessments (haematology, clinical chemistry and urinalysis).

### 2.7 Statistical methods

Sample size for this exploratory study was based on previous experience with similar studies, rather than on formal statistical considerations. The safety analysis set included all participants who received AZD4831 or placebo with available postdose safety data. The pharmacokinetic analysis set included all participants in the safety analysis set with evaluable pharmacokinetic data and no major protocol deviations. Analyses were performed using Statistical Analysis System (SAS) software, version 9.4. Descriptive statistics were not presented if there were fewer than 3 values available.

### 2.8 Nomenclature of targets and ligands

Key protein targets and ligands in this article are hyperlinked to corresponding entries in http://www.guidetopharmacology.org, the common portal for data from the IUPHAR/BPS Guide to PHARMACOLOGY,^25^ and are permanently archived in the Concise Guide to PHARMACOLOGY 2017/18.^26^, ^27^

## 3 RESULTS

### 3.1 Participant disposition and baseline characteristics

Forty white men, aged 19–50 years, were enrolled and randomized. The participants' mean BMI was 24.3 kg m^−2^ (standard deviation [SD] 2.3) in the AZD4831 group and 24.3 kg m^−2^ (SD 2.2) in the placebo group. All participants received one dose of AZD4831 (*n* = 30) or placebo (*n* = 10) and completed part A. All participants were included in the safety analyses and all participants who received AZD4831 were included in pharmacokinetic analyses. Four of the six participants in the AZD4831 45 mg cohort in part A also participated in part B of the study and all four completed part B. Two participants were withdrawn from the study before part B, with participant decision (*n* = 1) and noncompliance with the study protocol (*n* = 1) as the reasons for discontinuation.

### 3.2 Pharmacokinetic analyses

#### 3.2.1 Plasma pharmacokinetics of AZD4831 under fasted conditions

**Figure 1:**
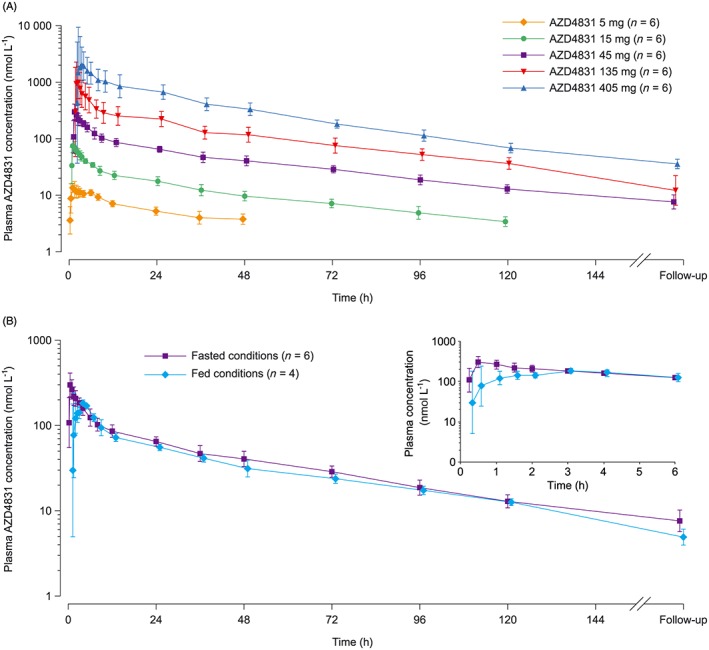
Plasma drug concentration *vs* time after single oral doses of AZD4831. A, Fasted conditions. B, Comparison of fasted and fed conditions (insert shows first 6 h after dose administration). Data points show the geometric mean and error bars show standard deviation

**Figure 2:**
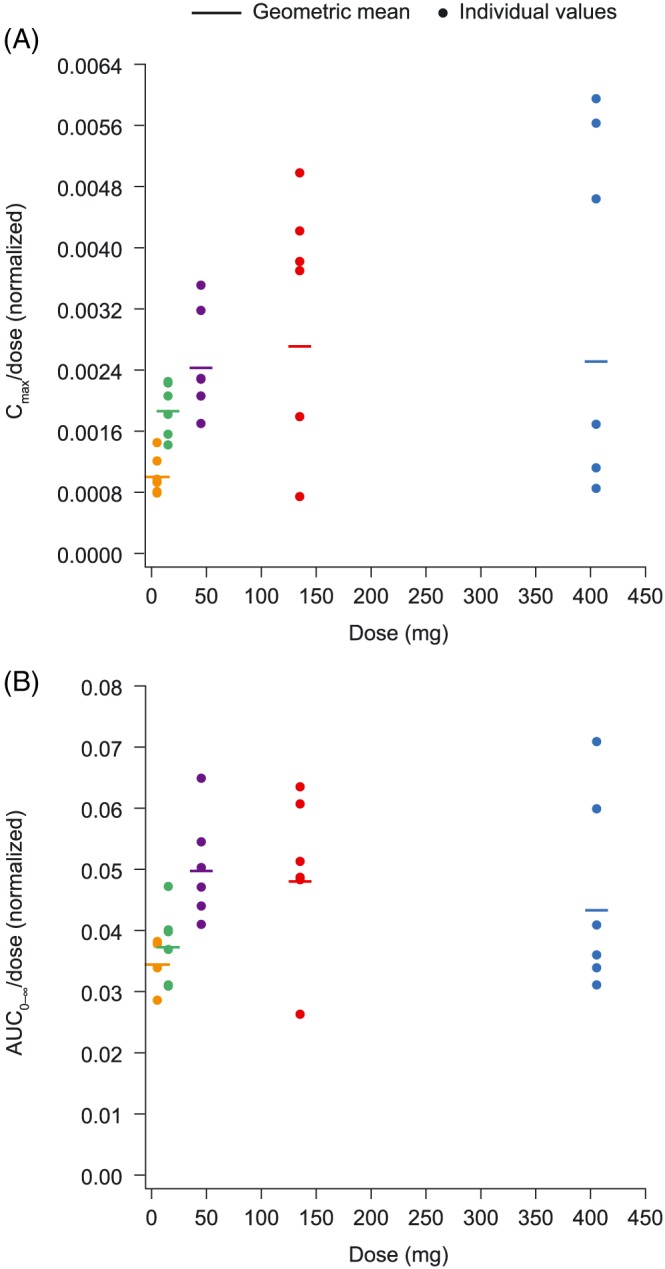
Dose‐normalized (A) C_max_ and (B) AUC_0–∞_ *vs* dose after oral administration of a single AZD4831 dose under fasted conditions. AUC_0–∞_ = area under the plasma concentration–time curve from time zero extrapolated to infinity; C_max_ = observed maximum concentration

AZD4831 distributed rapidly into plasma, reaching C_max_ at 0.51–1.00 hours after a single oral dose (Figure 1A; Table 1). AZD4831 had a long plasma half‐life, with *t*_1/2λz_ estimates of 38.2–50.0 hours. Plasma concentration of AZD4831 decreased in a biphasic manner. Across the entire dose range of 5 mg to 405 mg, AUC_0–∞_ increased proportionally with dose (slope estimate 1.060; 95% confidence interval [CI] 0.9943, 1.127), whereas C_max_ increases were slightly more than dose proportional (slope estimate 1.201; 95% CI 1.071, 1.332). When normalized to dose, AUC_0–∞_ and C_max_ were dose proportional in the range 45–405 mg and supra‐proportional below 45 mg (Figure 2). Coefficients of variation in AUC_0–∞_ and C_max_ were generally below 25% at doses of 45 mg or below (Table 1). Mean clearance rate and volume of distribution were similar across the entire dose range (Table 1).

**Table 1:** Summary of pharmacokinetic parameters of AZD4831 after single oral doses

**Parameter**	**Fasted**					**Fed**
	**5 mg (*n* = 6)**	**15 mg (*n* = 6)**	**45 mg (n = 6)**	**135 mg (n = 6)**	**405 mg (n = 6)**	**45 mg (*n* = 4)**
AUC_0–*t*_, h*nmol L^−1^	294.4 (13.2)	1444 (16.2)	6126 (15.1)	18 460 (31.9)	50 350 (35.3)	5365 (10.6)
AUC_0–∞_, h*nmol L^−1^	513.9 (13.6)	1669 (16.4)	6685 (16.5)	19 360 (32.5)	52 390 (34.3)	5790 (8.3)
C_max_, nmol L^−1^	14.96 (23.9)	83.42 (19.4)	326.1 (27.8)	1092 (83.1)	3037 (105.8)	183.6 (9.4)
*t*_max_, h	1.00 (0.50–5.98)	0.74 (0.50–1.00)	0.51 (0.50–1.02)	0.74 (0.50–1.50)	0.75 (0.50–2.00)	3.00 (2.00–4.00)
*t*_1/2λz_, h	37.6^a^ (28.9)	45.2 (9.7)	49.9 (6.8)	48.4 (14.2)	38.1 (7.5)	58.0 (15.1)
CL/F, L h^−1^	29.0^a^ (15.1)	26.9 (16.4)	20.1 (16.6)	20.8 (32.4)	23.1 (34.3)	23.2 (8.4)
CL_R_, L h^−1^	15.1 (21.1)	14.4 (12.9)	9.7 (13.6)	9.4 (14.2)	7.9 (62.1)	NE
V_z_/F, L	1579^a^ (17.4)	1751 (20.2)	1448 (12.9)	1454 (41.9)	1269 (40.4)	1944 (16.5)

Data are geometric mean (coefficient of variation), except for *t*_max_, which is shown as median (range).

a *n* = 4.

λz, elimination rate constant; AUC_0–∞_, area under the plasma concentration–time curve from time zero extrapolated to infinity; AUC_0–*t*_, area under the plasma concentration–time curve from time zero to time of last quantifiable analyte concentration; CL/F, apparent clearance; CL_R_, renal clearance; C_max_, observed maximum concentration; NE, not estimated; *t*_1/2λz_, half‐life associated with terminal slope (λz) of a semi‐logarithmic concentration–time curve; *t*_max_, time to reach maximum observed concentration; V_Z_/F, apparent volume of distribution.

#### 3.2.2 Effect of food intake on plasma pharmacokinetics of AZD4831

Administration of AZD4831 45 mg immediately after a high‐fat, high‐calorie meal led to a reduced rate of absorption compared with administration under fasting conditions (Figure 1B; Table 1). C_max_ was reduced by 44% and *t*_max_ was delayed by 2.5 hours. Food intake before dosing also slightly reduced AUC_0–∞_ and slightly increased *t*_1/2λz_ (Table 1).

#### 3.2.3 Urinary pharmacokinetics of AZD4831 under fasted conditions

The mean fraction of the AZD4831 dose excreted in urine over the first 48 hours was similar across the doses tested, ranging from 28.0% to 32.3%. Mean renal clearance ranged from 8.882 L h^−1^ (AZD4831 405 mg) to 15.42 L h^−1^ (AZD4831 5 mg) (Table 1).

### 3.3 Safety and tolerability

#### 3.3.1 Adverse events

In part A, 21 AEs in total were reported in 12/30 volunteers who received AZD4831 (40.0%) and 4/10 volunteers who received placebo (40.0%; Table 2). No AEs were reported in part B. All AEs were mild or moderate in intensity and no serious AEs or deaths occurred during the study. The most commonly reported AEs were headache (AZD4831, *n* = 4; placebo, *n* = 4) and maculopapular rash (AZD4831, *n* = 4; placebo, *n* = 0). The investigator judged that the AEs of headache, maculopapular rash and restlessness were possibly related to treatment. With the exception of maculopapular rash, the occurrence and intensity of AEs were similar between dose groups. All AEs of maculopapular rash were of moderate intensity. Maculopapular rash was reported by one participant in the 45 mg cohort, one in the 135 mg cohort and two in the 405 mg cohort. Of these, two received treatment with antihistamine tablets (cetirizine), one received treatment with topical methylprednisolone and one did not receive any treatment. The participant who experienced maculopapular rash in the AZD4831 45 mg cohort was withdrawn from the study before part B (with protocol noncompliance as the reason for discontinuation). AEs of maculopapular rash had a duration of 5–9 days and were reported 7–9 days after dosing; all had resolved by the end of the study.

**Table 2:** Adverse events

	**Fasted**							**Fed**
	**Pooled placebo**	**AZD4831**					**Pooled AZD4831**	**AZD4831**
	(*n* = 10)	**5 mg (*n* = 6)**	**15 mg (*n* = 6)**	**45 mg (*n* = 6)**	**135 mg (*n* = 6)**	**405 mg (*n* = 6)**	(*n* = 30)	**45 mg (*n* = 4)**
Any AE, *n* (%)	4 (40.0)	2 (33.3)	2 (33.3)	3 (50.0)	3 (50.0)	2 (33.3)	12 (40.0)	0
AEs by preferred term, n (%)								
Epistaxis	0	0	1 (16.7)	0	0	0	1 (3.3)	0
Gastroenteritis	0	0	0	1 (16.7)	0	0	1 (3.3)	0
Headache	4 (40.0)	0	1 (16.7)	2 (33.3)	1 (16.7)	0	4 (13.3)	0
Nasopharyngitis	0	0	0	0	1 (16.7)	0	1 (3.3)	0
Oropharyngeal pain	0	1 (16.7)	0	0	0	1 (16.7)	2 (6.7)	0
Rash maculopapular	0	0	0	1 (16.7)	1 (16.7)	2 (33.3)	4 (13.3)	0
Restlessness	1 (10.0)	1 (16.7)	0	0	0	0	1 (3.3)	0
Rhinitis	0	0	0	0	0	1 (16.7)	1 (3.3)	0

AE, adverse event.

#### 3.3.2 Laboratory and physical assessments

**Figure 3:**
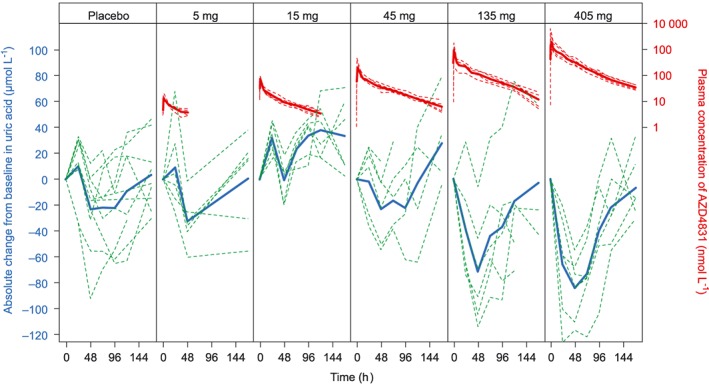
Change in serum uric acid concentration from baseline after single oral doses of ASD4831

There were no systemic or dose‐related changes in clinical chemistry (Table S1), haematology (Table S2) or urinary parameters, except for decreases in serum uric acid concentration associated with the administration of AZD4831 at doses of 135 mg or 405 mg (Figure 3). At 48 hours after dosing, mean serum uric acid concentrations had decreased by 71.77 μmol L^−1^ (95% CI 29.15, 114.39) for AZD4831 135 mg and by 84.42 μmol L^−1^ (95% CI 58.90, 109.94) for AZD4831 405 mg, compared with mean predose levels of 383.0 μmol L^−1^ (SD 47.5) and 329.0 μmol L^−1^ (SD 54.2), respectively (Table S1). There were no clinically significant changes in vital signs or electrocardiography parameters during the study (Table S3).

## 4 DISCUSSION

Myeloperoxidase inhibitors hold therapeutic potential as novel treatments for patients with cardiovascular diseases. Single oral doses of the myeloperoxidase inhibitor AZD4831 were rapidly absorbed and had a long half‐life in healthy men in this randomized, single‐blind, placebo‐controlled, phase I, first‐in‐human study. Administration of AZD4831 was generally well tolerated, with maculopapular rash being the only identified risk. These findings support the further clinical development of AZD4831.

AZD4831 reached its peak concentration in plasma within approximately 1 hour of oral dosing, and levels then decreased in a biphasic manner. Both AUC_0–∞_ and C_max_ increased approximately proportionally with dose, and the coefficients of variation were consistently low, indicating the predictable systemic delivery of oral AZD4831 in humans over the dose range tested (5–405 mg). The long plasma half‐life of AZD4831 (up to 50 hours) indicates that once‐daily dosing may provide sustained inhibition of myeloperoxidase. In vitro metabolism studies indicate that the cytochrome P450 (CYP) enzymes CYP3A4 and CYP3A5 are involved in the metabolism of AZD4831 (unpublished data), although glucuronidation and de‐acetylation are also likely to contribute to its overall metabolism. The administration of AZD4831 immediately after a high‐fat, high‐calorie meal resulted in a slower rate of absorption than administration after an overnight fast, but food intake did not have a substantial effect on overall AZD4831 exposure or plasma half‐life.

No serious AEs were reported during the study. An increased risk of opportunistic infections due to inhibition of myeloperoxidase in neutrophil granules was not anticipated at this level of AZD4831 exposure. Maculopapular rash was reported in four participants who received AZD4831 (13.3%), and was considered to be treatment related. All cases were moderate in intensity and resolved by the end of the study. Although the number of AEs reported was insufficient for the assessment of dose–response relationships for safety events, maculopapular rash was not reported in participants who received placebo or AZD4831 5 mg or 15 mg (the two lowest doses tested). No AEs were reported in participants who received AZD4831 45 mg under fed conditions, but this was only tested in four participants, none of whom experienced maculopapular rash in fasted conditions. Overall, maculopapular rash was identified as a potential risk for AZD4831, to be monitored in future studies. No other safety concerns were identified.

Clinical laboratory safety assessments revealed reductions in serum uric acid concentrations at the highest tested doses of AZD4831 (135 mg and 405 mg) in the exploratory pharmacodynamic assessments. Decreased uric acid levels have also been observed after the administration of another myeloperoxidase inhibitor,^12^ suggesting that this may be a class effect of myeloperoxidase inhibitors. In vitro*,* AZD4831 inhibits the ability of myeloperoxidase to oxidize xanthine to uric acid, and to oxidize uric acid to allantoin (E Michaëlsson et al., manuscript in preparation), in agreement with published studies indicating that xanthine interferes with production of hypochlorous acid by myeloperoxidase^21^ and that uric acid is a physiological substrate of myeloperoxidase.^21^, ^22^ Furthermore, AZD4831 does not inhibit uric acid transport via URAT1 (SLC22A12) and is not a substrate or an inhibitor of xanthine oxidase in vitro (E Michaëlsson et al., manuscript in preparation). These findings suggest that the most likely explanation for decreased serum uric acid levels in volunteers in the present study is inhibition of myeloperoxidase‐mediated xanthine oxidation by AZD4831. The magnitude of this effect is consistent with the canonical metabolic pathway via xanthine oxidase remaining unaffected by AZD4831.

Uric acid generation from purines is augmented by tissue hypoxia, and has been reported to be increased in the cardiac tissue of patients with heart failure.^13^ This suggests an alternative or additional potential mechanism for reduced uric acid levels following AZD4831 inhibition of myeloperoxidase. A reduction in vascular myeloperoxidase activity could lead to increased nitric oxide bioavailability, which may improve vascular endothelial function and tissue perfusion, 11 with a resulting decrease in hypoxia‐dependent uric acid generation from purines. Whether this mechanism contributed to the reduced uric acid levels seen in the current study in healthy volunteers is unclear, as is whether greater effects on uric acid would been seen in patients with hypoxia. The present study did not aim to investigate the mechanisms underlying the effects of AZD4831 in the exploratory pharmacodynamic assessments. The phase I multiple ascending dose study of AZD4831 in healthy volunteers (ClinicalTrials.gov identifier: NCT03136991) will provide additional data on uric acid levels over a 10‐day treatment period. Future studies will aim to investigate the mechanism of AZD4831‐mediated uric acid reduction by comparing functional and biochemical outcomes. A planned study in patients referred for cardiac catheterization (ClinicalTrials.gov identifier: NCT03611153) will assess the effect of AZD4831 on haemodynamics, exercise capacity and endothelial function.

The present study provided clear pharmacokinetic parameters and the first safety data for AZD4831 in healthy human volunteers. The following aspects of the study should be borne in mind when interpreting the results. The number of participants in the study was the smallest required to meet study objectives without unnecessarily placing healthy volunteers at potential risk from an investigational drug. The study design did not anticipate the long half‐life of AZD4831, which resulted in amendment of the sampling schedule after the first cohort, to include additional time points. Finally, the study was not designed to assess the pharmacodynamics of AZD4831, except as an exploratory objective.

This first study of AZD4831 in humans revealed that oral administration led to rapid and dose‐dependent absorption, with generally low interparticipant variability in systemic exposure. AEs were mild or moderate, with maculopapular rash identified for monitoring in future studies. These findings support the further clinical development of AZD4831 as a treatment for chronic, inflammatory cardiovascular diseases.

## COMPETING INTERESTS

This study was funded by AstraZeneca. L‐M Gan, M Lagerström‐Fermér, H Ericsson, K Nelander, E‐L Lindstedt, E Michaëlsson, M Kjaer, M Heijer and C Whatling are employees of AstraZeneca and may own stock or stock options. R Fuhr is an employee of PAREXEL. AstraZeneca provided funding to PAREXEL for the conduct of this study. Data underlying the findings described in this manuscript may be obtained in accordance with AstraZeneca's data sharing policy described at https://astrazenecagrouptrials.pharmacm.com/ST/Submission/Disclosure.

## CONTRIBUTORS

L.‐M.G. was involved in design of the study, overseeing its execution and analysis of data. M.L.‐F., H.E., K.N. and M.K. contributed to study design. E.‐L.L. contributed to study design and development of the protocol. E.M. and C.W. contributed to study design and analysis of data. M.H. was the clinical bioanalytical scientist responsible for setting up and monitoring the bioanalytical pharmacokinetic assays. R.F. was the principal investigator for the study and was involved in study design, protocol development, obtaining ethics committee approval, clinical study conduct, safety evaluations, dose escalation decisions and review of the study report. All authors contributed to interpretation of the data and writing or critical review of the manuscript.

## Supporting information


**Table S1** Clinical laboratory findings: Chemistry
**Table S2** Clinical laboratory findings: Haematology
**Table S3** Vital signs
