# Supplementary material for: Safety, tolerability, pharmacokinetics and effect on serum uric acid of the myeloperoxidase inhibitor AZD4831 in a randomized, placebo‐controlled, phase I study in healthy volunteers
Source: Br J Clin Pharmacol. 2019 Feb 18;85(4):762–70. doi: 10.1111/bcp.13855 (PMC6422671; doi:10.1111/bcp.13855)
Supplement: Supplementary file 1 — Table S1 Clinical laboratory findings: Chemistry Table S2 Clinical laboratory findings: Haematology Table S3 Vital signs [file BCP-85-762-s001.docx]

# Supplementary Material

#### Table S1. Clinical laboratory findings: chemistry.

|  | **Fasted** | | | | | | **Fed** |
| --- | --- | --- | --- | --- | --- | --- | --- |
| **Analyte** | **Pooled placebo** | **AZD4831** | | | | | **AZD4831** |
|  |  | **5 mg** | **15 mg** | **45 mg** | **135 mg** | **405 mg** | **45 mg** |
|  | **(n = 10)** | **(n = 6)** | **(n = 6)** | **(n = 6)** | **(n = 6)** | **(n = 6)** | **(n = 4)** |
| ALT, µkat L^–1^ |  |  |  |  |  |  |  |
| Baseline | 0.440 (0.177) | 0.458 (0.145) | 0.494 (0.201) | 0.379 (0.091) | 0.398 (0.119) | 0.359 (0.082) | 0.329 (0.068) |
| 24 h | 0.400 (0.153) | 0.397 (0.159) | 0.475 (0.222) | 0.329 (0.098) | 0.323 (0.137) | 0.338 (0.119) | 0.324 (0.125) |
| 48 h | 0.398 (0.160) | 0.405 (0.142) | 0.515 (0.255) | 0.319 (0.095) | 0.325 (0.191) | 0.351 (0.097) | 0.325 (0.119) |
| Follow up | 0.426 (0.157) | 0.407 (0.063) | 0.504 (0.193) | 0.324 (0.059) | 0.299 (0.129) | 0.346 (0.071) | 0.364 (0.151) |
| Albumin, g L^–1^ |  |  |  |  |  |  |  |
| Baseline | 45.5 (1.3) | 46.0 (1.6) | 46.4 (2.8) | 44.7 (1.6) | 47.2 (1.9) | 46.7 (2.3) | 42.7 (3.1) |
| 24 h | 42.8 (3.7) | 42.6 (2.4) | 43.2 (2.7) | 41.6 (2.4) | 43.4 (2.4) | 43.7 (2.3) | 41.2 (3.8) |
| 48 h | 42.4 (3.0) | 43.3 (2.1) | 44.2 (2.6) | 42.5 (2.7) | 43.9 (2.8) | 44.2 (2.7) | 42.1 (3.4) |
| Follow up | 45.4 (2.9) | 45.3 (1.3) | 45.2 (3.1) | 44.1 (2.6) | 46.0 (2.6) | 46.6 (2.7) | 44.1 (3.2) |
| ALP, µkat L^–1^ |  |  |  |  |  |  |  |
| Baseline | 1.227 (0.164) | 1.146 (0.213) | 1.270 (0.376) | 1.190 (0.285) | 1.311 (0.302) | 0.965 (0.196) | 1.045 (0.276) |
| 24 h | 1.147 (0.175) | 0.997 (0.210) | 1.165 (0.364) | 1.061 (0.228) | 1.128 (0.226) | 0.958 (0.139) | 0.993 (0.202) |
| 48 h | 1.118 (0.169) | 0.989 (0.186) | 1.159 (0.329) | 1.097 (0.257) | 1.175 (0.250) | 0.947 (0.186) | 1.004 (0.205) |
| Follow up | 1.240 (0.134) | 1.094 (0.227) | 1.240 (0.380) | 1.165 (0.282) | 1.239 (0.208) | 1.037 (0.190) | 1.103 (0.265) |
| AST, µkat L^–1^ |  |  |  |  |  |  |  |
| Baseline | 0.381 (0.111) | 0.472 (0.133) | 0.430 (0.105) | 0.347 (0.059) | 0.354 (0.054) | 0.401 (0.068) | 0.308 (0.023) |
| 24 h | 0.327 (0.082) | 0.370 (0.103) | 0.394 (0.098) | 0.293 (0.069) | 0.304 (0.076) | 0.363 (0.077) | 0.281 (0.048) |
| 48 h | 0.317 (0.083) | 0.357 (0.077) | 0.406 (0.116) | 0.288 (0.074) | 0.308 (0.101) | 0.366 (0.075) | 0.296 (0.053) |
| Follow up | 0.384 (0.106) | 0.413 (0.097) | 0.420 (0.085) | 0.356 (0.054) | 0.332 (0.064) | 0.398 (0.062) | 0.358 (0.049) |
| Bilirubin, µmol L^–1^ |  |  |  |  |  |  |  |
| Baseline | 15.1 (7.1) | 12.3 (4.6) | 12.7 (7.7) | 13.0 (2.6) | 13.5 (4.6) | 15.4 (2.5) | 8.6 (1.7) |
| 24 h | 14.6 (4.8) | 12.3 (2.6) | 14.5 (6.3) | 12.0 (3.5) | 12.2 (4.3) | 14.9 (2.3) | 9.7 (0.6) |
| 48 h | 13.1 (3.6) | 10.6 (2.3) | 13.6 (6.0) | 12.3 (3.3) | 11.6 (3.5) | 13.4 (2.1) | 9.5 (1.3) |
| Follow up | 11.9 (4.6) | 12.5 (3.3) | 11.3 (5.5) | 12.9 (5.6) | 13.1 (5.1) | 13.2 (3.1) | 9.6 (2.3) |
| CRP, mg L^–1^ |  |  |  |  |  |  |  |
| Baseline | 1.11 (1.52) | 0.56 (0.32) | 0.77 (0.40) | 1.15 (1.13) | 0.85 (0.58) | 0.93 (0.92) | 0.62 (0.38) |
| 24 h | 0.71 (0.70) | 0.38 (0.22) | 0.53 (0.28) | 0.77 (0.71) | 0.60 (0.30) | 1.17 (1.28) | 0.42 (0.20) |
| 48 h | 0.66 (0.56) | 0.48 (0.40) | 0.62 (0.40) | 0.58 (0.49) | 0.50 (0.26) | 1.19 (1.17) | 0.42 (0.22) |
| Follow up | 0.67 (0.53) | 1.27 (1.43) | 1.37 (0.47) | 2.89 (5.30) | 8.94 (12.69) | 1.17 (0.86) | 2.35 (3.10) |
| Calcium, mmol L^–1^ |  |  |  |  |  |  |  |
| Baseline | 2.40 (0.06) | 2.43 (0.06) | 2.41 (0.15) | 2.36 (0.06) | 2.44 (0.07) | 2.38 (0.08) | 2.31 (0.06) |
| 24 h | 2.38 (0.08) | 2.36 (0.04) | 2.35 (0.11) | 2.34 (0.05) | 2.36 (0.08) | 2.39 (0.06) | 2.30 (0.10) |
| 48 h | 2.35 (0.08) | 2.38 (0.04) | 2.37 (0.10) | 2.34 (0.05) | 2.37 (0.07) | 2.40 (0.06) | 2.33 (0.07) |
| Follow up | 2.42 (0.08) | 2.42 (0.04) | 2.38 (0.14) | 2.35 (0.04) | 2.36 (0.11) | 2.43 (0.10) | 2.34 (0.08) |
| Creatinine, µmol L^–1^ |  |  |  |  |  |  |  |
| Baseline | 78.4 (5.0) | 86.5 (12.2) | 77.4 (9.6) | 73.3 (7.9) | 87.2 (7.5) | 80.4 (13.8) | 76.7 (8.0) |
| 24 h | 79.3 (8.6) | 82.2 (10.3) | 76.2 (10.7) | 75.8 (7.2) | 83.6 (8.7) | 86.2 (14.7) | 71.2 (11.4) |
| 48 h | 77.3 (6.3) | 80.9 (4.7) | 75.9 (12.3) | 73.2 (6.4) | 81.0 (9.1) | 81.1 (13.0) | 70.9 (9.3) |
| Follow up | 78.2 (5.9) | 84.8 (9.1) | 78.4 (9.8) | 74.6 (9.2) | 85.1 (12.7) | 81.1 (12.2) | 74.6 (5.0) |
| GGT, µkat L^–1^ |  |  |  |  |  |  |  |
| Baseline | 0.330 (0.063) | 0.308 (0.087) | 0.565 (0.261) | 0.344 (0.074) | 0.358 (0.178) | 0.320 (0.142) | 0.320 (0.053) |
| 24 h | 0.301 (0.054) | 0.288 (0.103) | 0.504 (0.232) | 0.315 (0.072) | 0.318 (0.171) | 0.298 (0.146) | 0.310 (0.053) |
| 48 h | 0.299 (0.049) | 0.287 (0.105) | 0.501 (0.243) | 0.312 (0.064) | 0.325 (0.161) | 0.290 (0.133) | 0.303 (0.051) |
| Follow up | 0.328 (0.066) | 0.307 (0.093) | 0.534 (0.262) | 0.300 (0.052) | 0.306 (0.143) | 0.298 (0.137) | 0.316 (0.087) |
| GFR, mL min^–1^ 1.73m^–2^ |  |  |  |  |  |  |  |
| Screening | 104 (6) | 98 (14) | 115 (16) | 110 (14) | 104 (15) | 103 (11) | 110 (14) |
| Follow up | 105 (7) | 98 (15) | 114 (12) | 112 (12) | 104 (22) | 104 (12) | 109 (15) |
| Glucose, mmol L^–1^ |  |  |  |  |  |  |  |
| Baseline | 4.95 (0.32) | 4.90 (0.39) | 4.81 (0.28) | 5.07 (0.65) | 5.16 (0.31) | 4.64 (0.27) | 5.17 (0.40) |
| 24 h | 4.97 (0.26) | 4.69 (0.32) | 4.57 (0.21) | 4.90 (0.29) | 4.74 (0.58) | 4.77 (0.30) | 5.06 (0.38) |
| 48 h | 4.89 (0.28) | 4.76 (0.32) | 4.72 (0.23) | 5.06 (0.19) | 4.75 (0.37) | 4.88 (0.35) | 5.27 (0.13) |
| Follow up | 4.75 (0.41) | 4.78 (0.68) | 4.62 (0.45) | 4.84 (0.54) | 4.71 (0.54) | 4.63 (0.39) | 5.18 (0.25) |
| Phosphate, mmol L^–1^ |  |  |  |  |  |  |  |
| Baseline | 1.04 (0.10) | 1.12 (0.10) | 1.10 (0.15) | 1.20 (0.11) | 1.08 (0.20) | 1.09 (0.13) | 1.30 (0.09) |
| 24 h | 1.07 (0.13) | 1.12 (0.09) | 1.14 (0.14) | 1.17 (0.08) | 1.06 (0.23) | 1.07 (0.11) | 1.10 (0.08) |
| 48 h | 1.01 (0.15) | 0.99 (0.12) | 1.11 (0.16) | 1.12 (0.09) | 1.01 (0.19) | 1.06 (0.15) | 1.09 (0.05) |
| Follow up | 1.13 (0.13) | 1.11 (0.10) | 1.15 (0.08) | 1.12 (0.20) | 1.00 (0.21) | 1.12 (0.17) | 1.08 (0.11) |
| Potassium, mmol L^–1^ |  |  |  |  |  |  |  |
| Baseline | 4.33 (0.34) | 4.16 (0.24) | 4.20 (0.29) | 4.12 (0.18) | 4.24 (0.16) | 4.10 (0.28) | 4.26 (0.15) |
| 24 h | 4.38 (0.20) | 4.14 (0.23) | 4.12 (0.24) | 4.29 (0.10) | 4.11 (0.11) | 4.42 (0.31) | 4.28 (0.26) |
| 48 h | 4.34 (0.26) | 4.23 (0.16) | 4.23 (0.25) | 4.18 (0.20) | 4.06 (0.17) | 4.33 (0.33) | 4.27 (0.05) |
| Follow up | 4.46 (0.27) | 4.32 (0.22) | 4.39 (0.30) | 4.18 (0.18) | 4.07 (0.20) | 4.39 (0.18) | 4.34 (0.20) |
| Sodium, mmol L^–1^ |  |  |  |  |  |  |  |
| Baseline | 138.3 (2.5) | 136.8 (1.1) | 137.8 (1.7) | 138.7 (1.4) | 139.9 (1.9) | 138.9 (1.4) | 139.9 (2.4) |
| 24 h | 138.4 (1.9) | 136.3 (0.8) | 137.5 (1.0) | 139.2 (1.7) | 139.8 (0.7) | 139.2 (1.0) | 139.6 (1.6) |
| 48 h | 138.6 (1.3) | 137.5 (0.6) | 138.8 (0.8) | 140.9 (2.2) | 140.3 (1.4) | 139.6 (1.0) | 140.1 (1.4) |
| Follow up | 138.2 (2.0) | 138.4 (1.5) | 136.9 (1.1) | 139.5 (1.3) | 140.2 (2.1) | 141.5 (2.6) | 139.6 (0.6) |
| Urate, µmol L^–1^ |  |  |  |  |  |  |  |
| Baseline | 328.5 (53.9) | 325.9 (61.3) | 332.8 (25.0) | 359.8 (109.2) | 383.0 (47.5) | 329.0 (54.2) | 321.5 (64.8) |
| 24 h | 338.5 (36.8) | 335.1 (75.2) | 364.7 (35.3) | 361.7 (111.9) | 344.3 (18.6) | 262.8 (65.4) | 296.4 (62.8) |
| 48 h | 305.2 (33.8) | 293.2 (64.0) | 331.6 (30.7) | 339.8 (113.0) | 311.3 (18.6) | 244.6 (51.2) | 283.0 (57.0) |
| 72 h | 315.3 (33.1) | ND | 355.7 (25.4) | 362.1 (111.6) | 339.2 (13.9) | 256.1 (44.7) | 313.2 (47.1) |
| 96 h | 314.9 (43.4) | ND | 366.5 (25.3) | 366.3 (112.8) | 346.2 (25.0) | 289.3 (46.2) | 316.0 (47.3) |
| 120 h | 327.6 (39.0) | ND | 370.8 (40.4) | 382.6 (93.3) | 365.9 (42.1) | 307.2 (50.9) | 316.5 (67.1) |
| Follow up | 328.7 (51.6) | 326.3 (84.2) | 366.3 (47.1) | 397.6 (112.6) | 370.3 (31.4) | 322.2 (59.1) | 314.0 (77.9) |
| Urea, mmol L^–1^ |  |  |  |  |  |  |  |
| Baseline | 4.89 (0.92) | 4.93 (1.17) | 4.07 (0.77) | 4.18 (0.49) | 4.78 (0.94) | 4.26 (1.01) | 4.44 (1.17) |
| 24 h | 4.44 (0.59) | 4.66 (0.90) | 4.35 (0.61) | 4.25 (0.47) | 4.50 (0.56) | 4.55 (1.01) | 4.04 (0.45) |
| 48 h | 4.85 (0.77) | 4.52 (1.08) | 4.30 (0.74) | 4.24 (0.55) | 4.24 (0.74) | 4.68 (1.07) | 4.19 (0.41) |
| Follow up | 4.52 (1.07) | 4.57 (1.29) | 4.79 (1.46) | 3.90 (0.63) | 4.15 (1.35) | 3.87 (0.96) | 4.18 (0.78) |

Data are mean (standard deviation).

Direct bilirubin and indirect bilirubin not shown.

ALT, alanine aminotransferase; ALP, alkaline phosphatase; AST, aspartate aminotransferase; CRP, C reactive protein; GGT, γ-glutamyl transferase; GFR, glomerular filtration rate; ND, not determined.

#### Table S2. Clinical laboratory findings: haematology.

|  | **Fasted** | | | | | | **Fed** |
| --- | --- | --- | --- | --- | --- | --- | --- |
| **Analyte** | **Pooled placebo** | **AZD4831** | | | | | **AZD4831** |
|  |  | **5 mg** | **15 mg** | **45 mg** | **135 mg** | **405 mg** | **45 mg** |
|  | **(n = 10)** | **(n = 6)** | **(n = 6)** | **(n = 6)** | **(n = 6)** | **(n = 6)** | **(n = 4)** |
| Basophils, 10^9^ L^–1^ |  |  |  |  |  |  |  |
| Baseline | 0.03 (0.01) | 0.04 (0.02) | 0.04 (0.01) | 0.06 (0.03) | 0.03 (0.01) | 0.04 (0.01) | 0.03 (0.02) |
| 24 h | 0.03 (0.01) | 0.03 (0.01) | 0.04 (0.01) | 0.04 (0.02) | 0.02 (0.01) | 0.04 (0.01) | 0.03 (0.01) |
| 48 h | 0.03 (0.02) | 0.04 (0.02) | 0.04 (0.01) | 0.04 (0.02) | 0.03 (0.01) | 0.03 (0.01) | 0.03 (0.02) |
| Follow up | 0.04 (0.02) | 0.04 (0.01) | 0.04 (0.02) | 0.05 (0.02) | 0.03 (0.01) | 0.04 (0.01) | 0.04 (0.01) |
| Eosinophils, 10^9^ L^–1^ |  |  |  |  |  |  |  |
| Baseline | 0.16 (0.08) | 0.29 (0.10) | 0.26 (0.10) | 0.23 (0.12) | 0.14 (0.06) | 0.25 (0.18) | 0.28 (0.20) |
| 24 h | 0.15 (0.08) | 0.25 (0.12) | 0.21 (0.07) | 0.20 (0.12) | 0.11 (0.07) | 0.18 (0.15) | 0.19 (0.10) |
| 48 h | 0.16 (0.08) | 0.23 (0.10) | 0.22 (0.07) | 0.21 (0.10) | 0.12 (0.07) | 0.24 (0.26) | 0.20 (0.15) |
| Follow up | 0.17 (0.08) | 0.26 (0.12) | 0.22 (0.08) | 0.20 (0.15) | 0.14 (0.09) | 0.27 (0.27) | 0.18 (0.12) |
| Erythrocyte MCH concentration, g L^–1^ | | |  |  |  |  |  |
| Baseline | 339 (9) | 339 (6) | 335 (6) | 329 (10) | 350 (9) | 346 (6) | 346 (6) |
| 24 h | 342 (9) | 340 (5) | 341 (2) | 335 (8) | 354 (5) | 345 (8) | 343 (14) |
| 48 h | 341 (11) | 338 (6) | 340 (7) | 335 (15) | 353 (7) | 347 (7) | 343 (8) |
| Follow up | 343 (8) | 334 (5) | 334 (2) | 333 (10) | 350 (11) | 345 (5) | 347 (11) |
| Erythrocyte MCH, pg |  |  |  |  |  |  |  |
| Baseline | 30.3 (1.6) | 29.9 (1.1) | 29.3 (0.9) | 28.3 (2.1) | 30.7 (0.3) | 31.2 (1.2) | 30.5 (1.4) |
| 24 h | 30.4 (1.7) | 29.8 (1.2) | 29.6 (0.7) | 28.4 (2.1) | 31.0 (0.4) | 30.8 (1.5) | 30.2 (2.0) |
| 48 h | 30.3 (1.8) | 29.6 (1.0) | 29.5 (1.1) | 28.4 (2.3) | 30.9 (0.4) | 31.0 (1.6) | 30.2 (1.7) |
| Follow up | 30.6 (1.4) | 29.5 (1.1) | 29.1 (1.0) | 28.3 (2.3) | 30.8 (0.5) | 31.0 (1.1) | 30.7 (1.0) |
| Erythrocyte MCV, fL |  |  |  |  |  |  |  |
| Baseline | 89.5 (3.3) | 88.1 (2.8) | 87.5 (2.0) | 86.0 (6.0) | 87.7 (2.0) | 90.3 (3.3) | 88.2 (5.2) |
| 24 h | 88.8 (3.3) | 87.5 (3.3) | 86.8 (2.0) | 84.8 (5.8) | 87.5 (1.9) | 89.4 (3.8) | 88.0 (5.6) |
| 48 h | 88.9 (2.9) | 87.7 (3.1) | 86.9 (2.9) | 84.9 (5.9) | 87.4 (1.6) | 89.4 (3.7) | 88.2 (5.8) |
| Follow up | 89.2 (2.9) | 88.2 (3.1) | 87.3 (2.5) | 85.0 (5.8) | 88.2 (1.7) | 89.8 (3.6) | 88.3 (5.1) |
| Erythrocytes, 10^12^ L^–1^ |  |  |  |  |  |  |  |
| Baseline | 5.03 (0.41) | 5.02 (0.36) | 5.26 (0.28) | 5.26 (0.22) | 4.97 (0.34) | 4.90 (0.26) | 4.68 (0.16) |
| 24 h | 4.91 (0.53) | 4.90 (0.28) | 5.08 (0.27) | 5.01 (0.18) | 4.69 (0.26) | 4.81 (0.31) | 4.74 (0.15) |
| 48 h | 4.84 (0.49) | 4.88 (0.26) | 5.03 (0.23) | 5.02 (0.33) | 4.68 (0.30) | 4.67 (0.22) | 4.89 (0.11) |
| Follow up | 4.90 (0.43) | 4.98 (0.36) | 5.06 (0.37) | 5.08 (0.35) | 4.62 (0.25) | 4.83 (0.35) | 4.72 (0.16) |
| Haematocrit, ratio |  |  |  |  |  |  |  |
| Baseline | 0.45 (0.02) | 0.44 (0.02) | 0.46 (0.02) | 0.45 (0.02) | 0.44 (0.03) | 0.44 (0.01) | 0.41 (0.01) |
| 24 h | 0.44 (0.04) | 0.43 (0.02) | 0.44 (0.03) | 0.42 (0.03) | 0.41 (0.02) | 0.43 (0.01) | 0.42 (0.03) |
| 48 h | 0.43 (0.03) | 0.43 (0.02) | 0.44 (0.02) | 0.43 (0.03) | 0.41 (0.03) | 0.42 (0.01) | 0.43 (0.03) |
| Follow up | 0.44 (0.03) | 0.44 (0.02) | 0.44 (0.03) | 0.43 (0.02) | 0.41 (0.02) | 0.43 (0.02) | 0.42 (0.04) |
| Haemoglobin, g L^–1^ |  |  |  |  |  |  |  |
| Baseline | 152 (8) | 150 (6) | 154 (11) | 149 (7) | 153 (10) | 153 (6) | 142 (3) |
| 24 h | 149 (12) | 146 (6) | 150 (9) | 142 (9) | 145 (8) | 148 (6) | 143 (9) |
| 48 h | 146 (10) | 145 (7) | 148 (8) | 142 (8) | 144 (9) | 145 (5) | 148 (9) |
| Follow up | 149 (10) | 147 (8) | 147 (10) | 143 (7) | 143 (9) | 149 (8) | 145 (9) |
| Leukocytes, 10^9^ L^–1^ |  |  |  |  |  |  |  |
| Baseline | 6.23 (1.40) | 5.83 (0.76) | 5.89 (1.52) | 6.09 (0.80) | 5.65 (1.27) | 5.62 (0.67) | 5.78 (1.35) |
| 24 h | 6.01 (2.03) | 5.43 (0.71) | 5.94 (1.90) | 5.28 (1.15) | 5.11 (1.13) | 5.53 (1.12) | 5.51 (0.85) |
| 48 h | 5.52 (1.51) | 5.74 (1.20) | 6.08 (1.88) | 5.69 (1.06) | 5.28 (1.23) | 5.53 (1.14) | 5.93 (1.07) |
| Follow up | 6.05 (1.17) | 5.94 (1.03) | 5.81 (1.43) | 5.88 (1.09) | 6.80 (3.32) | 5.84 (1.41) | 6.37 (1.60) |
| Lymphocytes, 10^9^ L^–1^ |  |  |  |  |  |  |  |
| Baseline | 1.80 (0.66) | 1.79 (0.29) | 1.94 (0.63) | 2.00 (0.37) | 1.90 (0.33) | 2.05 (0.46) | 1.88 (0.47) |
| 24 h | 1.68 (0.47) | 1.62 (0.37) | 1.75 (0.55) | 1.74 (0.42) | 1.52 (0.40) | 1.40 (0.19) | 1.76 (0.61) |
| 48 h | 1.71 (0.63) | 1.60 (0.38) | 1.71 (0.49) | 1.79 (0.36) | 1.66 (0.47) | 1.51 (0.30) | 1.85 (0.77) |
| Follow up | 1.78 (0.51) | 1.47 (0.34) | 1.63 (0.38) | 1.61 (0.45) | 1.51 (0.26) | 1.43 (0.19) | 1.79 (0.37) |
| Monocytes, 10^9^ L^–1^ |  |  |  |  |  |  |  |
| Baseline | 0.43 (0.11) | 0.39 (0.08) | 0.48 (0.15) | 0.45 (0.09) | 0.42 (0.13) | 0.42 (0.10) | 0.41 (0.15) |
| 24 h | 0.41 (0.12) | 0.37 (0.06) | 0.46 (0.17) | 0.37 (0.13) | 0.35 (0.12) | 0.34 (0.09) | 0.34 (0.16) |
| 48 h | 0.40 (0.10) | 0.38 (0.06) | 0.52 (0.21) | 0.38 (0.10) | 0.37 (0.11) | 0.40 (0.14) | 0.40 (0.16) |
| Follow up | 0.40 (0.07) | 0.45 (0.15) | 0.49 (0.07) | 0.45 (0.13) | 0.48 (0.32) | 0.40 (0.08) | 0.39 (0.12) |
| Neutrophils, 10^9^ L^–1^ |  |  |  |  |  |  |  |
| Baseline | 3.71 (1.37) | 3.18 (0.49) | 3.07 (1.08) | 3.24 (0.78) | 3.02 (0.94) | 2.73 (0.35) | 3.10 (0.90) |
| 24 h | 3.62 (1.82) | 3.07 (0.47) | 3.37 (1.36) | 2.83 (0.90) | 3.00 (0.79) | 3.47 (0.79) | 3.11 (0.79) |
| 48 h | 3.11 (1.11) | 3.38 (1.02) | 3.47 (1.39) | 3.16 (0.95) | 3.00 (0.83) | 3.22 (0.62) | 3.35 (1.20) |
| Follow up | 3.56 (1.10) | 3.59 (0.73) | 3.32 (1.20) | 3.45 (1.25) | 4.46 (2.99) | 3.54 (1.02) | 3.85 (1.22) |
| Platelets, 10^9^ L^–1^ |  |  |  |  |  |  |  |
| Baseline | 235 (53) | 245 (40) | 202 (19) | 226 (34) | 212 (32) | 193 (28) | 227 (60) |
| 24 h | 224 (46) | 246 (44) | 193 (28) | 220 (39) | 200 (25) | 190 (23) | 227 (50) |
| 48 h | 222 (53) | 240 (50) | 204 (25) | 222 (44) | 202 (29) | 187 (20) | 233 (51) |
| Follow up | 249 (69) | 249 (38) | 221 (33) | 245 (38) | 218 (24) | 205 (21) | 250 (62) |
| Reticulocytes, 10^9^ L^–1^ |  |  |  |  |  |  |  |
| Baseline | 75.4 (7.6) | 82.7 (15.2) | 85.3 (23.3) | 75.7 (16.0) | 76.0 (24.7) | 69.1 (13.7) | 82.5 (9.8) |
| 24 h | 71.3 (10.0) | 76.9 (15.3) | 77.2 (18.6) | 69.8 (16.4) | 72.4 (19.9) | 68.5 (16.4) | 77.4 (5.4) |
| 48 h | 68.8 (8.3) | 84.4 (20.3) | 78.1 (24.5) | 77.3 (19.5) | 70.1 (15.1) | 66.3 (20.0) | 84.5 (7.9) |
| Follow up | 88.1 (12.0) | 91.6 (15.7) | 89.9 (32.9) | 79.1 (19.3) | 82.8 (19.3) | 78.6 (19.4) | 82.9 (10.8) |

Data are mean (standard deviation).

MCH, mean corpuscular haemoglobin; MCV, mean corpuscular volume.

#### Table S3. Vital signs.

|  | **Fasted** | | | | | | **Fed** |
| --- | --- | --- | --- | --- | --- | --- | --- |
|  | **Pooled placebo** | **AZD4831** | | | | | **AZD4831** |
|  |  | **5 mg** | **15 mg** | **45 mg** | **135 mg** | **405 mg** | **45 mg** |
|  | **(n = 10)** | **(n = 6)** | **(n = 6)** | **(n = 6)** | **(n = 6)** | **(n = 6)** | **(n = 4)** |
| SBP, mmHg |  |  |  |  |  |  |  |
| Baseline | 116 (11) | 117 (11) | 119 (8) | 125 (13) | 117 (6) | 116 (6) | 122 (14) |
| 0.5 h | 118 (7) | 116 (10) | 115 (7) | 122 (13) | 113 (8) | 116 (9) | 121 (15) |
| 1 h | 118 (11) | 114 (8) | 115 (8) | 125 (12) | 110 (5) | 114 (6) | 122 (16) |
| 2 h | 117 (12) | 119 (10) | 113 (4) | 122 (17) | 113 (10) | 114 (9) | 120 (12) |
| 3 h | 117 (8) | 119 (12) | 112 (5) | 120 (14) | 113 (7) | 112 (6) | 125 (13) |
| 4 h | 117 (8) | 118 (9) | 113 (5) | 121 (13) | 116 (8) | 115 (5) | 123 (17) |
| 6 h | 116 (8) | 118 (11) | 119 (9) | 120 (9) | 113 (10) | 113 (4) | 120 (7) |
| 8 h | 118 (12) | 117 (9) | 116 (7) | 116 (11) | 113 (8) | 113 (5) | 121 (14) |
| 12 h | 117 (11) | 118 (13) | 119 (5) | 124 (12) | 119 (9) | 114 (8) | 124 (8) |
| 24 h | 115 (10) | 112 (5) | 113 (4) | 118 (9) | 115 (7) | 113 (4) | 114 (9) |
| 36 h | 118 (10) | 119 (6) | 118 (5) | 121 (9) | 118 (12) | 118 (4) | 127 (9) |
| 48 h | 116 (10) | 116 (8) | 114 (7) | 120 (10) | 114 (10) | 118 (4) | 116 (7) |
| DBP, mmHg |  |  |  |  |  |  |  |
| Baseline | 72 (6) | 71 (9) | 68 (5) | 73 (14) | 73 (5) | 70 (11) | 73 (14) |
| 0.5 h | 74 (8) | 71 (7) | 68 (4) | 73 (14) | 69 (5) | 70 (7) | 66 (16) |
| 1 h | 73 (7) | 71 (7) | 67 (4) | 72 (14) | 70 (4) | 69 (8) | 65 (11) |
| 2 h | 70 (9) | 71 (5) | 65 (2) | 69 (15) | 68 (6) | 68 (6) | 66 (18) |
| 3 h | 72 (8) | 72 (9) | 65 (3) | 70 (13) | 68 (6) | 67 (5) | 69 (11) |
| 4 h | 72 (8) | 71 (7) | 66 (6) | 69 (16) | 69 (6) | 69 (7) | 67 (15) |
| 6 h | 69 (8) | 69 (8) | 65 (2) | 66 (7) | 66 (6) | 66 (5) | 70 (15) |
| 8 h | 72 (9) | 68 (5) | 64 (2) | 68 (15) | 70 (6) | 67 (5) | 71 (17) |
| 12 h | 68 (8) | 69 (9) | 64 (4) | 68 (11) | 69 (7) | 67 (4) | 69 (15) |
| 24 h | 69 (8) | 69 (5) | 67 (2) | 71 (11) | 69 (3) | 71 (2) | 69 (12) |
| 36 h | 68 (7) | 69 (4) | 63 (4) | 66 (14) | 67 (6) | 68 (4) | 71 (15) |
| 48 h | 69 (6) | 70 (4) | 66 (5) | 69 (11) | 71 (5) | 71 (7) | 73 (13) |
| Pulse, bpm |  |  |  |  |  |  |  |
| Baseline | 54 (7) | 61 (5) | 61 (4) | 59 (9) | 60 (8) | 56 (6) | 55 (6) |
| 0.5 h | 55 (5) | 61 (4) | 58 (9) | 59 (11) | 59 (6) | 61 (8) | 63 (6) |
| 1 h | 55 (4) | 61 (5) | 61 (9) | 57 (10) | 61 (9) | 61 (8) | 66 (11) |
| 2 h | 56 (10) | 60 (4) | 58 (6) | 59 (10) | 59 (7) | 58 (8) | 64 (9) |
| 3 h | 57 (6) | 60 (4) | 58 (4) | 60 (8) | 59 (6) | 57 (6) | 64 (9) |
| 4 h | 58 (7) | 64 (5) | 59 (8) | 58 (8) | 60 (7) | 60 (8) | 62 (10) |
| 6 h | 66 (6) | 68 (5) | 68 (9) | 61 (10) | 67 (8) | 63 (8) | 64 (8) |
| 8 h | 60 (6) | 62 (5) | 63 (11) | 59 (11) | 62 (4) | 59 (7) | 59 (9) |
| 12 h | 66 (9) | 68 (4) | 69 (10) | 63 (12) | 70 (5) | 63 (8) | 63 (10) |
| 24 h | 58 (6) | 59 (5) | 62 (7) | 58 (11) | 65 (9) | 60 (7) | 57 (9) |
| 36 h | 66 (10) | 68 (5) | 73 (9) | 66 (11) | 73 (7) | 65 (5) | 62 (9) |
| 48 h | 58 (6) | 64 (9) | 65 (12) | 64 (13) | 67 (9) | 65 (9) | 57 (4) |

Data are mean (standard deviation)

bpm, beats per minute; DBP, diastolic blood pressure; SBP, systolic blood pressure.
